# Effect of an Antibacterial Polysaccharide Produced by *Chaetomium globosum* CGMCC 6882 on the Gut Microbiota of Mice

**DOI:** 10.3390/foods10051084

**Published:** 2021-05-13

**Authors:** Xincheng Sun, Zichao Wang, Xuyang Hu, Chengxin Zhao, Xiaogen Zhang, Huiru Zhang

**Affiliations:** 1 Henan Key Laboratory of Cold Chain Food Quality and Safety Control, Zhengzhou University of Light Industry, Zhengzhou 450001, China; sunxinch@zzuli.edu.cn (X.S.); xyhu@zzuli.edu.cn (X.H.); chxzhao@zzuli.edu.cn (C.Z.); xgzhang@zzuli.edu.cn (X.Z.); 2 Collaborative Innovation Center of Food Production and Safety, Zhengzhou 450001, China; 3 College of Biological Engineering, Henan University of Technology, Zhengzhou 450001, China; zhr@haut.edu.cn

**Keywords:** polysaccharide, antibacterial activity, toxicity, body health, gut microbiota

## Abstract

Previously, a polysaccharide produced by *Chaetomium* *globosum* CGMCC 6882 was found to have antibacterial activity, but its toxic effects on body health and gut microbiota were concealed. Recent results showed that this polysaccharide was safe to Caco-2 cells and mice, while it reduced the body weight gain of mice from 10.5 ± 1.21 g to 8.4 ± 1.17 g after 28 days administration. Acetate, propionate, butyrate and total short-chain fatty acids concentrations increased from 23.85 ± 1.37 μmol/g, 10.23 ± 0.78 μmol/g, 7.15 ± 0.35 μmol/g and 41.23 ± 0.86 μmol/g to 42.77 ± 1.29 μmol/g, 20.03 ± 1.44 μmol/g, 12.06 ± 0.51 μmol/g and 74.86 ± 2.07 μmol/g, respectively. Furthermore, this polysaccharide enriched the abundance of gut microbiota and the *Firmicutes*/*Bacteroidetes* ratio was increased from 0.5172 to 0.7238. Overall, this study provides good guidance for the promising application of polysaccharides as preservatives in foods and in other fields in the future.

## 1. Introduction

The intestinal tract is home to a large number of complex and diverse gut microbiota [1], such as different types of bacteria, viruses and fungi [2]. As a bridge between diet and host health, gut microbiota not only affects the digestion and absorption of nutrients in the diet, but also regulates the normal physiological functions and the occurrence of diseases in the host [3]. Recently, with the help of next-generation high-throughput sequencing technology, bioinformatics and metagenomics [4], researchers have verified that gut microbiota is vital to host health, and the disruption of gut microbiota has been shown to be associated with multiple diseases, including metabolic syndrome [5], obesity [6], tumor [7], diabetes [8], HIV [9], flu [10], fatigue [11], brain health [12], etc. At the same time, intestinal flora transplantation has shown promising application prospects in the treatment of diseases [13].

With their antibacterial and growth-promoting properties, antibiotics are widely used in disease treatment and daily production. However, abuse of antibiotics not only increases the antibiotic residue in foods and the resistance of disease-fighting microorganisms [14], but it also leads to drug-resistant genes being transmitted from livestock and microorganisms to humans [15]. Meanwhile, antibiotics can directly damage body health via disrupting the homeostasis of gut microbiota in the intestinal tract. For example, Cox et al. [16] found that mice treated with continuous low doses of penicillin could develop a higher body weight due to the disruption of the gut microbiota. Zhang et al. [2] reported that the gene expression and metabolic homeostasis of mice were affected by the administration of perfluorooctane sulfonate. Xu et al. [17] demonstrated that antibiotics could promote tumor initiation in mice by inducing gut microbiota dysbiosis. Therefore, looking for a new generation of safe, high-efficient, widely applicable and non-toxic antibiotics has been growing increasingly important.

As a kind of macromolecule connected by more than ten monosaccharides through a glycoside bond, the gut microbiota could convert polysaccharides into short-chain fatty acids (SCFAs), such as acetic, propionic and butyric acid, thus having a positive effect on gut microbiota and body health [18]. However, the effects of antibacterial polysaccharides on body health, especially the gut microbiota in the intestinal tract, are concealed and poorly understood. Herein, this work assayed the toxicity of an antibacterial polysaccharide (GCP) produced by *Chaetomium globosum* CGMCC 6882 [19] to Caco-2 cells. Secondly, the effects of GCP on the body weight and serum biochemistry of normal mice were detected. Finally, the influence of GCP on the gut microbiota of normal mice was assessed. We hope that this work could provide some help and guidance for the application of bacteriostatic polysaccharides.

## 2. Materials and Methods

### 2.1. Preparation of GCP

The preparation of GCP produced from *C.* *globosum* CGMCC 6882 was based on the methods reported in our previous work [20]. Briefly, fermentation liquid was filtered and centrifuged at 12,000× *g* for 30 min to remove mycelium and cells. The supernatant was de-proteinized by adding three volumes Sevag solution, then three volumes cold alcohol were added and it was kept at 4 °C overnight to precipitate GCP. The crude GCP re-dissolved in distilled water was de-pigmented with AB-8 macroporous resin (Beijing NuoqiYa Biotechnology Co., Ltd., Beijing, China) and then dialyzed for 48 h in distilled water. After this, GCP solution was filtered with a 0.22 μm filter and applied to a Sepharose CL-6B column (2.5 cm × 60 cm) for further purification, eluted with 0.1 mol/L NaCl solution at a flow rate of 0.6 mL/min, and the fraction was then collected. In the end, the purified GCP was lyophilized for further experiments.

### 2.2. Cell Viability Assay

The toxicity of GCP to Caco-2 cells (American Type Culture Collection, ATCC, HTB037) was measured by the 3-(4,5-Dimethylthiazol-2-yl)-2,5-bromo diphenyltetrazolium (MTT) assay reported in our previous work [21] with some modifications. Dimethyl sulfoximine (DMSO), Dulbecco’s modified Eagle medium (DMEM) and MTT were brought from Sigma-Aldrich (Shanghai, China). Meanwhile, fetal bovine serum, penicillin and streptomycin were brought from Sangon Biotech (Shanghai, China). The Caco-2 cells were cultured in DMEM containing 10% (*v*/*v*) fetal bovine serum, 100 U/mL penicillin and 100 μg/mL of streptomycin at 37 °C in a humidified 5% CO_2_ incubator (Series 8000 WJ, Thermo Fisher Seientific, Waltham, MA, USA). Before experiment, the dried GCP powder was dissolved in different concentrations of DMEM solution (100–600 μg/mL) and DMEM solution without GCP was used as the control. Briefly, Caco-2 cells were seeded into 96-well plates at a concentration of 2 × 10^4^ cells/mL and incubated at 37 °C in 5% CO_2_ for 24 h before treatment. 

Then, 100 μL GCP at different concentrations was added into wells and cultured for another 24 h. Afterwards, 20 μL of 5 mg/mL MTT was added. After 4 h of incubation, cell supernatant was discarded and 150 μL DMSO was added to dissolve the insoluble crystals in the cell. In the end, the absorbance of each well was recorded by a microplate reader (Bio-Rad Laboratories, Inc., Pleasanton, CA, USA) at 490 nm.

### 2.3. Experimental Design and Samples Collection

Specific pathogen free-male mice (20 ± 1 g) were purchased from the Laboratory Animal Center of Henan province (SCXK: 2017-0002; Zhengzhou, China). All mice were held in independent cages and kept in specific pathogen-free conditions at temperatures of 24 ± 1 °C, humidity of 60 ± 5%, and with a light to dark cycle of 12 h/12 h. During experiments, all mice were monitored every day, and the experiments were performed strictly according to the guidelines for the care and use of laboratory animals (Henan University of Technology, Zhengzhou, China). Forty mice were randomly divided into four groups (*n* = 10) after adaption for 7 days. One group was used as the normal control group (NC), and another three groups were designed as the experimental group and treated with 100 μg/mL GCP (low-dose group), 200 μg/mL GCP (middle-dose group) and 400 μg/mL GCP (high-dose group), respectively. Then, mice in the experimental groups were orally administered 0.5 mL GCP once a day, and mice in the normal control group were administered equal distilled water. The animal experiments lasted for 28 days and all mice were weighted weekly. During the whole experiment, all mice had free access to a basic diet and distilled water. At the end of experiment, all mice were killed after fasting for 12 h. Blood samples were collected from the orbit and centrifuged at 3000 r/min for 10 min to collect the serum. Meanwhile, the contents of the cecum were immediately collected in plastic tubes (1.5 mL) and stored at −80 °C for further analyses.

### 2.4. Serum Biochemical Index Detection

The levels of aspartate transaminase, alanine aminotransferase, total protein, albumin, globulin, urea, high-density lipoprotein and low-density lipoprotein, and the glucose concentration in the serum, were tested using the serum analyzer (BS-420, Shenzhen Mindray Biomedical Electronics Co., Ltd., Wuhan, China).

### 2.5. Measurement of SCFAs

The concentration of SCFAs in cecum contents was analyzed according to the method of Wu et al. [22] with some modifications. Briefly, 50 mg of cecum contents was suspended in 500 μL of saturated sodium chloride solution and vortexed uniformly for 30 min. Then, the solutions were acidified with 20 μL of 10% H_2_SO_4_ and extracted with 1 mL ethyl ether. After this, the mixtures were centrifuged at 12,000× *g* and 4 °C for 10 min, and the obtained organic layer of supernatant was mixed with 0.25 g of anhydrous sodium sulfate for 5 min to remove water. In the end, the supernatant was filtered with 0.22 μm organic-based filter membrane and the SCFAs in the organic layer were analyzed by a 7890A GC system (Agilent Technologies Inc., Santa Clara, CA, USA) equipped with a flame ionization detector; the carrier gas was N_2_, the shunt ratio was 20:1 and the flow rate was 1.5 mL/min. The chromatographic column was HP-INNOWAX (Agilent, 30 m × 0.25 mm × 0.25 μm), and the temperature procedure was as follows: temperature was increased from 60 °C to 190 °C at 20 °C/min and maintained for 4 min. The injection temperature was 200 °C, the ionization temperature was 250 °C and the injection volume was 5 μL. The standard curve was made by the external standard method and the concentrations of SCFAs were calculated according to the standard curve.

### 2.6. DNA Extraction of Cecum Contents and High-Throughput Sequencing

The cecum contents were sent to Majorbio Co., Ltd., China (Shanghai, China) for DNA extraction and sequencing of 16S rRNA gene. Briefly, the total microbial DNA was extracted from cecum contents (*n* = 10) with the DNA extraction kit and tested by agarose gel electrophoresis. The V3-V4 hypervariable region of 16S rRNA was amplified by polymerase chain reaction (PCR), and the primer sequences were 338F (5′-ACTCCTACGGGAGGCAGCAG-3′) and 806R (5′-CTCCTACGGGAGG CAGCAG-3′). Then, the PCR products of equimolar concentrations were sequenced using Illumina MiSeq platforms according to the operation manual.

### 2.7. Statistical Analysis

Data were expressed as the mean ± standard deviation (SD). Data were subjected to one-way ANOVA, and significance differences were analyzed using SPSS version 19.0 (IBM Company, Armonk, NY, USA).

## 3. Results and Discussion

### 3.1. Cell Viability Assay

**Figure 1:**
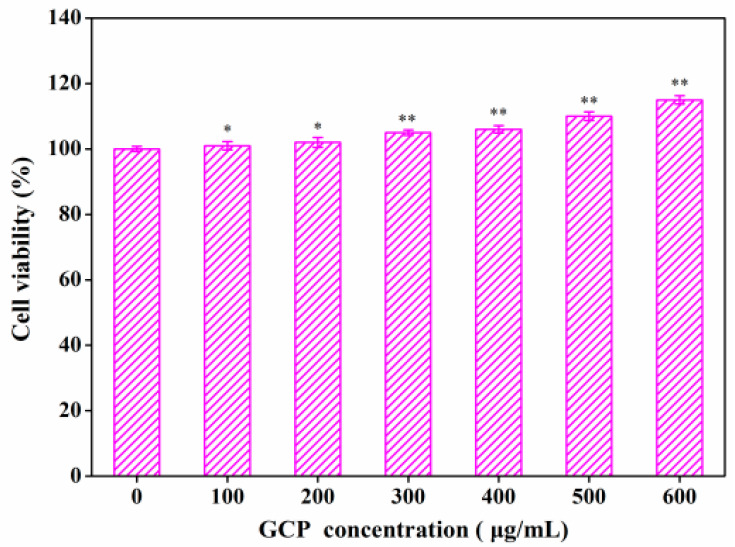
Cell viability assay of GCP to Caco-2 cells (*n* = 5). Significance was determined through ANOVA, * *p* < 0.05, ** *p* < 0.01.

Antibiotics easily cause some side effects in the treatment of diseases, such as antibiotic-associated diarrhea [23]. Even though it is the bacteriocin approved by JECFA, nisin could also bring some adverse effects to the gut microbiota of the body [24]. As shown in Figure 1, with the experimental concentrations of GCP from 100 μg/mL to 600 μg/mL, cell viability increased from 101 ± 0.8% to 115 ± 1.3% (*p* < 0.05), suggesting that GCP was not toxic to Caco-2 cells. This toxicity result was similar to that of *Ganoderma lucidum* polysaccharide reported in our earlier work [21] and in works by other researchers. For example, Zhang et al. [25] found that the alkali-soluble polysaccharides from *Arctium lappa* L. had no toxicity to RAW264.7 cells. Caillot et al. [26] reported that the blackberry wine polysaccharides had no toxicity to RAW 264.7 macrophages. Meanwhile, during the whole experiment, there were no signs of disease or death in mice, indicating the security of GCP to mice. Furthermore, He et al. [27] demonstrated that a novel polysaccharide produced by *Streptomyces Virginia* H03 was safe to mice when administered at doses of 500 mg/kg/day. Therefore, the concentrations of GCP used in the following tests were 100 μg/mL, 200 μg/mL and 400 μg/mL, respectively.

### 3.2. Effect of GCP on the Body Weight of Normal Mice

As can be seen from Table 1, the administration of GCP decreased the average body weight of mice, and the body weight gain of mice significantly (*p* < 0.05) decreased from 10.5 ± 1.21 g to 8.4 ± 1.17 g. Chen et al. [23] reported that *Pueraria lobata* polysaccharide could effectively reduce the average body weight of mice. Meanwhile, Tian et al. [28] and Yin et al. [6] demonstrated that *Lycium ruthenicum* and resveratrol had similar weight loss effects as those seen in this work. However, Yang et al. [29] and Wei et al. [30] found that flaxseed polysaccharides and *Musa basjoo* had almost no effects on the weight loss of mice, which was inconsistent with the results of this work. The weight gain control of GCP might relate to the metabolism of acetate, alanine aminotransferase and aspartate aminotransferase [6,28], and this will be analyzed in the following work.

### 3.3. Effect of GCP on the Serum Biochemistry of Normal Mice

The effects of GCP on physiological parameters in the serum of mice are shown in Table 2. There was a decreasing trend (*p* < 0.05) in the concentration of aspartate transaminase and alanine aminotransferase in the mice serum after the administration of GCP; the aspartate transaminase concentration decreased from 38.5 ± 3.37 U/L to 35.4 ± 1.87 U/L, and the alanine aminotransferase concentration reduced from 121.5 ± 6.43 U/L to 105.4 ± 10.91 U/L. Meanwhile, Guo et al. [31] and Yin et al. [6] reported that the weight loss effects of nanobubble water and resveratrol were partly related to the reduction inaspartate transaminase and alanine aminotransferase in the body, which could partly explain why the GCP reduced the weight of the mice in Table 1. However, the activities of total protein, albumin, globulin, urea, high-density lipoprotein, low-density lipoprotein and the glucose in the mice serum among the four groups underwent little change, suggesting that GCP had no toxicity to mice.

### 3.4. Effect of GCP on the SCFAs of Normal Mice

**Figure 2:**
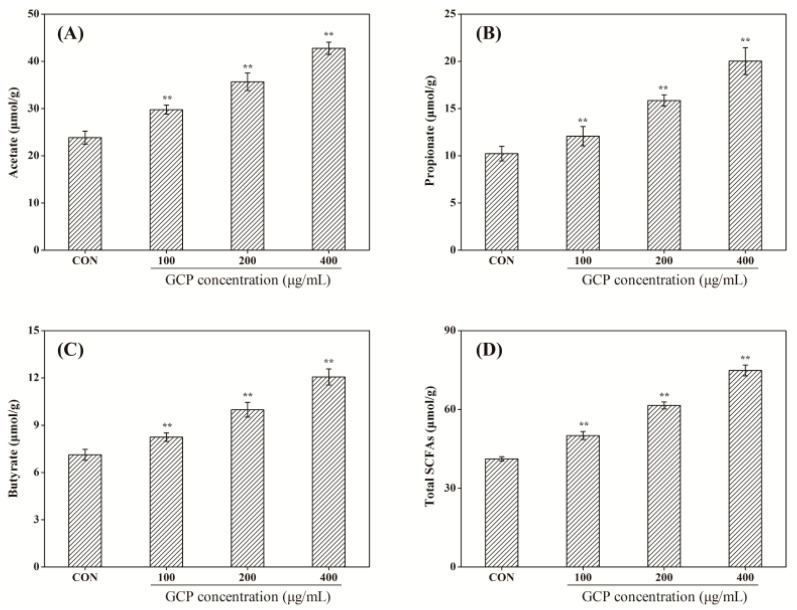
Effect of GCP on the concentrations of acetate, propionate, butyrate and total SCFAs in the cecum of mice (*n* = 10). (**A**) Acetate, (**B**) propionate, (**C**) butyrate, (**D**) total SCFAs. CON: control group. Significance was determined through ANOVA, ** *p* < 0.01.

Due to the glycoside bonds between monosaccharides and their complex structure, most polysaccharides are resistant to saliva and gastric and small intestinal juices, which are further utilized by gut microbiota to produce SCFAs [18,32]. As illustrated in Figure 2, the concentrations of SCFAs in the control group were significantly lower than in experimental groups (*p* < 0.01). After administration of 400 μg/mL GCP for 28 days, the acetate concentration in the cecum contents of mice increased from 23.85 ± 1.37 μmol/g to 42.77 ± 1.29 μmol/g, the propionate concentration increased from 10.23 ± 0.78 μmol/g to 20.03 ± 1.44 μmol/g, the butyrate concentration increased from 7.15 ± 0.35 μmol/g to 12.06 ± 0.51 μmol/g, and the total SCFAs increased from 41.23 ± 0.86 μmol/g to 74.86 ± 2.07 μmol/g. SCFAs could inhibit the growth and reproduction of pathogenic bacteria by reducing the acidity of the intestinal environment, but could also produce a positive systematic physiological effect on the host via regulating the innate and adaptive immune systems and intestinal permeability [33]. Furthermore, SCFAs may also be conducive to losing weight by promoting satiety [34], especially acetate, thus explaining the reduction in body weight gain caused by GCP in Table 1. Many factors affect the utilization of polysaccharides by gut microbiota, such as linkage mode, chain type, molecular weight, sulfate content, etc. [35]. In future work, we will investigate the effect of the GCP’s structure on the gut microbiota utilization.

### 3.5. Effect of GCP on the Composition and Diversity of Gut Microbiota

#### 3.5.1. Diversity Analysis of the Structure of Gut Microbiota

Through α-diversity analysis, a series of statistical analysis indexes was used to estimate and reflect the abundance and diversity of microbial communities. Sobs, ACE and Chao index reflected the species richness of operational taxonomic units (OTUs) in the gut microbiota, while the Simpson and Shannon indexes reflected the differences in species diversity in the gut microbiota [22]. As shown in Table 3, the Sobs, ACE, Chao and Shannon index of the gut microbiota in the experimental groups showed an increasing trend (*p* < 0.05) in a concentration-dependent manner compared to the control group, and the Simpson index showed a downward trend (*p* < 0.05). This indicates that GCP increased the species richness and diversity of the gut microbiota in the cecum contents of mice.

**Figure 3:**
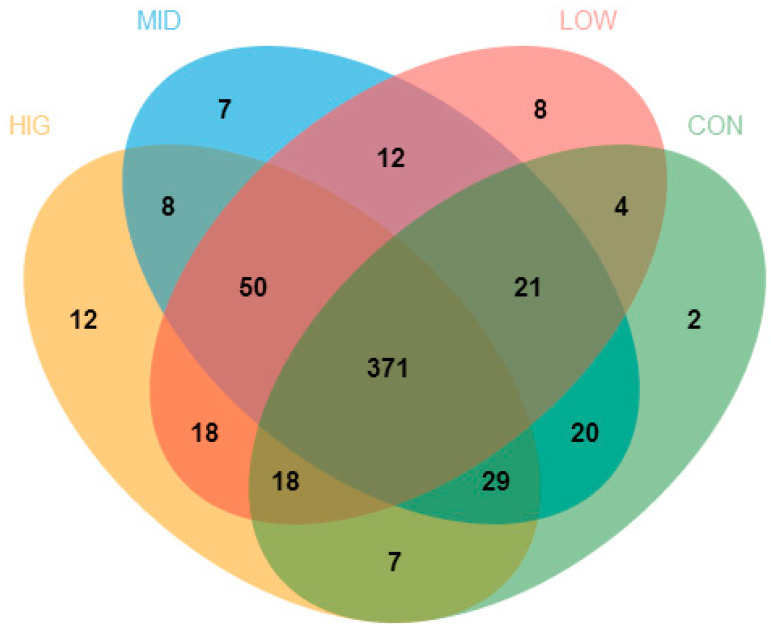
Venn diagram of colon gut microbiota (*n* = 10). CON: control group; LOW: 100 μg/mL GCP; MID: 200 μg/mL GCP; HIG: 400 μg/mL GCP.

The different numbers of OTUs are illustrated among the four groups by the Venn diagram in (Figure 3). Among all the OTUs in this work, 371 were shared by all groups. Meanwhile, the numbers of OTUs shared by experimental groups and control group were 414 (low-dose group), 441 (middle-dose group) and 425 (high-dose group). Furthermore, 88 OTUs were detected in the low-dose group but not in the control group, and 77 and 88 OTUs were detected separately in the middle-dose group and high-dose group. Furthermore, a different group had its own separate set of OTUs: 2 in the control group, 8 in the low-dose group, 7 in the middle-dose group and 12 in the high-dose group. However, the total numbers of OTUs in the control group, low-dose group, middle-dose group and high-dose group were 472, 502, 518 and 513, respectively. This suggested that GCP increased the species richness of the gut microbiota in the cecum of mice.

#### 3.5.2. Composition Analysis of the Gut Microbiota

**Figure 4:**
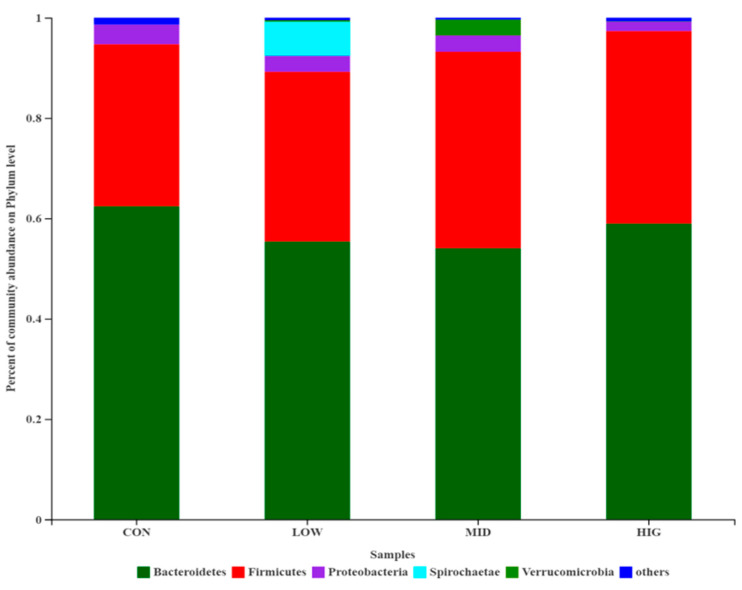
Relative abundance of the gut microbiota at the phylum level (*n* = 10). CON: control group; LOW: 100 μg/mL GCP; MID: 200 μg/mL GCP; HIG: 400 μg/mL GCP.

The relative abundance of cecum gut microbiota composition of each group is shown in Figure 4. At the phylum level, the gut microbiota in the four groups mainly consisted of *Bacteroidetes*, *Firmicutes*, *Proteobacteria*, *Spirochaetae* and *Verrucomicrobia*, and these five phyla in all groups represented approximately ≥95% of the sequences. The relative abundances of *Bacteroidetes* in the four groups were 62.41%, 55.38%, 54.05% and 58.97%, and those of *Firmicutes* were 32.28%, 33.81%, 39.12% and 38.36%. Notably, th *Firmicutes*/*Bacteroidetes* (F/B) ratio increased from 0.5172 in the control group to 0.6105 (low-dose group), 0.7238 (middle-dose group) and 0.6505 (high-dose group), showing an increasing trend. The increased dose of GCP might have an effect on the change in ratio of F/B in mice gut microbiota, but as the two main communities that affect energy metabolism homeostasis [36], researchers have different opinions on the functions of *Firmicutes* and *Bacteroidetes*. Wu et al. [22] found that *Cyclocarya* *paliurus* polysaccharides alleviated the liver inflammation of mice by increasing the F/B ratio in their gut microbiota. However, Yang et al. [29] demonstrated that *Linum usitatissimum* L. polysaccharides mitigated the high-fat diet-induced metabolic syndrome in mice, which did not affect the relative abundance of *Bacteroidetes*, but reduced the relative abundance of *Firmicutes*. At the same time, a few researchers have suggested that obesity is not associated with the ratio of F/B [37,38]. The utilization and digestion of polysaccharides by gut microbiota are affected by many factors, including monosaccharide composition, linkage mode, chain type, molecular weight, sulfate content, etc. [4,29]. In future work, we will investigate the effect of high-dose GCP with a relatively low F/B ratio (0.6505), compared with the 0.7238 of the middle-dose group.

## 4. Conclusions

The toxicity of antibacterial polysaccharides to the body and gut microbiota is poorly understood. The results in the present work show that the antibacterial polysaccharide of GCP was safe for Caco-2 cells and mice. Meanwhile, GCP reduced the body weight gain of mice and increased the SCFAs concentration in the colon. Furthermore, GCP increased the diversity of gut microbiota and the *Firmicutes*/*Bacteroidetes* ratio. In future work, the specific bacteria in the microbiota of a gut affected by GCP, especially *Firmicutes* and *Bacteroidetes*, will be investigated.

## Figures and Tables

**Table 1:** Effect of GCP on the body weight of mice (*n* = 10).

Mice Weight	Control Group	GCP Concentration (μg/mL)		
		100	200	400
0 day (g)	20.2 ± 1.57 ^a^	20.4 ± 1.38 ^a^	20.0 ± 2.23 ^a^	20.8 ± 2.42 ^a^
7 day (g)	23.7 ± 2.27 ^b^	23.5 ± 2.09 ^b^	22.9 ± 3.37 ^b^	22.5 ± 2.45 ^b^
14 day (g)	26.5 ± 2.34 ^c^	26.1 ± 1.99 ^c^	25.7 ± 2.54 ^c^	25.4 ± 2.08 ^c^
21 day (g)	28.1 ± 2.14 ^d^	27.9 ± 2.23 ^d^	27.4 ± 2.15 ^d^	26.9 ± 2.64 ^d^
28 day (g)	30.7 ± 2.61 ^e^	30.1 ± 2.45 ^e^	29.1 ± 2.38 ^e^	29.2 ± 3.17 ^e^
Weight gain (g)	10.5 ± 1.21 ^a^	9.7 ± 1.09 ^b^	9.1 ± 0.88 ^c^	8.4 ± 1.17 ^d^

Different letters (a–e) represent significant differences, *p* < 0.05.

**Table 2:** Effect of GCP on the serum biochemistry of mice (*n* = 10).

Serum Biochemistry	Control Group	GCP Concentration (μg/mL)		
		100	200	400
Aspartate transaminase (U/L)	38.5 ± 3.37 ^a^	37.3 ± 2.98 ^a^	36.2 ± 3.29 ^b^	35.4 ± 1.87 ^b^
Alanine aminotransferase (U/L)	121.5 ± 6.43 ^a^	115.3 ± 9.31 ^b^	111.9 ± 11.34 ^c^	105.4 ± 10.91 ^d^
Total protein (g/L)	53.5 ± 3.04 ^a^	54.5 ± 5.09 ^a^	54.8 ± 4.10 ^a^	53.9 ± 2.08 ^a^
Albumin (g/L)	31.7 ± 1.63 ^a^	32.1 ± 3.14 ^a^	31.9 ± 2.68 ^a^	31.2 ± 2.10 ^a^
Globulin (g/L)	20.8 ± 1.41 ^a^	21.1 ± 2.15 ^a^	20.9 ± 3.32 ^a^	20.7 ± 2.47 ^a^
Urea (mmol/L)	11.62 ± 0.83 ^a^	11.75 ± 1.22 ^a^	11.69 ± 0.91 ^a^	11.66 ± 1.13 ^a^
High density lipoprotein (mmol/L)	1.66 ± 0.03 ^a^	1.68 ± 0.02 ^a^	1.63 ± 0.03 ^a^	1.65 ± 0.06 ^a^
Low density lipoprotein (mmol/L)	0.11 ± 0.008 ^a^	0.09 ± 0.004 ^a^	0.12 ± 0.012 ^a^	0.10 ± 0.007 ^a^
Glucose (mmol/L)	5.74 ± 0.41 ^a^	5.84 ± 0.12 ^a^	5.76 ± 0.51 ^a^	5.79 ± 0.32 ^a^

Different letters (a–d) represent significant differences, *p* < 0.05.

**Table 3:** Effect of GCP on the α-diversity of gut microbiota (*n* = 10).

Diversity Index	Control Group	GCP Concentration (μg/mL)		
		100	200	400
Sobs	360.66 ± 18.64 ^a^	368.66 ± 15.21 ^b^	382.00 ± 21.35 ^c^	379.50 ± 26.33 ^c^
ACE	401.41 ± 29.32 ^a^	408.41 ± 35.03 ^b^	416.06 ± 27.06 ^c^	414.54 ± 38.09 ^c^
Chao1	404.96 ± 25.54 ^a^	418.25 ± 34.17 ^b^	426.88 ± 29.13 ^c^	424.96 ± 34.10 ^c^
Simpson	0.074 ± 0.09 ^a^	0.057 ± 0.01 ^b^	0.049 ± 0.05 ^c^	0.043 ± 0.09 ^d^
Shannon	3.84 ± 0.19 ^a^	3.94 ± 0.27 ^b^	4.19 ± 0.31 ^c^	4.30 ± 0.39 ^d^

Different letters (a–d) represent significant differences, *p* < 0.05.

## Data Availability

Not applicable.
